# Excluding autism or excluding everything? The problem of broad definitions in the England and Wales Draft Mental Health Bill

**DOI:** 10.1192/bjb.2023.60

**Published:** 2024-06

**Authors:** Peter Beazley

**Affiliations:** 1 University of East Anglia, Norwich, UK

**Keywords:** Psychiatry and law, autism spectrum disorders, in-patient treatment, comorbidity, consent and capacity

## Abstract

The recent Draft Mental Health Bill for England and Wales proposes changes to the Mental Health Act 1983 which will include, for the first time, a legal definition of autism. This article explores the specific potential issue that the definition, owing to its breadth, potentially encompasses a number of conditions other than autism, consequently leaving the definitionally dependent concept of ‘psychiatric disorder’ significantly narrowed in scope. The potential implications of this – primarily the concern that a range of other conditions and presentations could be unintentionally excluded from the scope of the civil powers in the Mental Health Act – are discussed.

We recently wrote^1^ about a range of unintended consequences that potentially arise as a consequence of the proposed changes to legislation contained in the Draft Mental Health Bill for England and Wales^2^ (henceforth ‘draft bill’). Within that review, we commented briefly on the potential breadth of the definition of autism adopted and remarked that this could have the potential to be problematic. The present article seeks to expand on this specific point, in particular considering the practical issues that arise from attaching an exclusionary function to such a broad definition. Although these proposed changes are specific to England and Wales, the potential implications are much broader, given that the social and political pressures that give rise to these changes (a desire to reduce the scope of deprivations of liberty and restrictive interventions for people with learning disabilities and autism) are likely to be common to many jurisdictions.

The proposed definition of autism in the draft bill is ‘a lifelong developmental disorder of the mind that affects how people perceive, communicate and interact with others’. This term is identical to that contained in the National Strategy for Autism in England^3^ and is very similar to the definition adopted by the National Autistic Society,^4^ though in both of these cases the term ‘developmental disability’ is used in preference to ‘developmental disorder’. This is the first time that a legal definition of autism has been created in England and Wales, with even the Autism Act (2009) avoiding a formal definition and simply using the undefined term ‘autism spectrum conditions’ (the explanatory notes for the Autism Act, s.1(11), clarify that this was a deliberate decision on account of the breadth of the ‘spectrum’ of autism, which could change over time in response to ‘research and experience’). Compared with a clinical definition of autism as given in either of the two major clinical classification systems, the proposed definition is much broader and therefore in practice quite different, omitting any quantification or qualification of functional impairment, severity or indeed other common features of autism, including restricted interests or repetitive patterns of behaviour. For these reasons, we suggest that it is necessary to differentiate the concept of autism as proposed in the draft bill from the more widely understood clinical concept; therefore, we refer to the proposed definition as ‘legal autism’.

Understanding what is meant by ‘legal autism’ is important, because the bill proposes an amendment such that people who have legal autism (or indeed a learning disability) would be excluded from the scope of key provisions of the Mental Health Act; specifically, s.3, which regulates the process of admission for treatment. This is achieved by replacing the previous broad category of mental disorder (defined in s.1(2)) with a new category of ‘psychiatric disorder’, which is defined as ‘any mental disorder other than autism or learning disability’ (c.1(3)). An admission under s.3 would therefore only be possible if the ‘nature’ or ‘degree’ of the condition that gives rise to the need for detention is derived from this more restricted concept of ‘psychiatric disorder’ (schedule 1, c.2). Although a time-limited detention for assessment under s.2 could still occur (allowing a period of 28 days for assessment), as could a detention under the parts of the Act that relate to the detention and treatment of people linked to the criminal justice system (i.e. part III), a potentially indefinite detention under s.3 would no longer be possible. This would be, without doubt, a significant change to the status quo. This is particularly so given that the concept of ‘psychiatric disorder’ is definitionally dependent on the concept of legal autism (logically, anything that is legal autism cannot be considered ‘psychiatric disorder’ in terms of the stated definitions). It follows that the broader the definition of ‘autism’, the narrower the scope of the concept of ‘psychiatric disorder’.

The key concern is that although the definition of legal autism works well for an inclusionary purpose – i.e. it is broad enough that it would encompass all people with ‘clinical autism’ – this same breadth is problematic when it is then used in an exclusionary way as proposed. Specifically, it is the author's view that this definition could, at face value, be applied to a range of conditions. For instance, the term ‘developmental disorder’ – arguably the key clinical component within the proposed definition – has been used within reputable scholarly literature to describe a range of conditions including alcoholism^5^ and addiction,^6^ anxiety,^7^ bipolar disorder,^8^ schizophrenia,^9–11^ Alzheimer's disease^12,13^ and personality disorder (including psychopathy^14–16^ and borderline and antisocial personality disorders^17^). Indeed, a paper by Wu and Barnes^15^ concerning psychopathy directly replicates the first half of the legal definition, i.e. ‘psychopathy is a developmental disorder of the mind’. With regard to the other components, the qualifier ‘of the mind’ does not apply any obvious distinction or differentiation between autism and other conditions. The final element, ‘perceive, communicate and interact with others’ perhaps warrants more consideration but would again be likely to be non-specific to autism. For instance, all of the psychotic disorders would be considered to clinically influence perception, and, quite possibly because of this impact, people with psychosis would be expected to demonstrate impairments in both communication and social functioning. Similarly, personality disorder could be argued to influence perceptions (particularly around interpersonal communication and the intent of others), and undoubtedly most personality disorder presentations would inherently show some impact on both communication and interactions with others.

Could such disorders therefore be considered indistinct from ‘legal autism’ and hence excluded from most civil powers of the Act? At face value, this suggestion might seem absurd, and it would certainly be a significant unintended side-effect if true in practice. It is therefore an important consideration even if the probability of such a consequence arising is low. The remainder of the present article attempts to consider whether this is simply an academic concern or one that could genuinely have adverse practical consequences, commencing with a review of some of the most obvious reasons for dismissing these concerns.

## Does the qualifier ‘lifelong’ provide a limitation to ‘developmental disorder’ that would in practice be specific to autism?

One starting point might be to respond that the term ‘lifelong’, in its adjectival form, provides a suitable limitation specific to autism and would therefore prevent such a scenario arising. But the power of this argument depends largely on what one actually means by ‘lifelong’. The definition in the *Oxford English Dictionary* (‘[l]asting or continuing for a lifetime, or throughout one's life’) arguably doesn't provide much guidance. Taking a narrow view, does it, for instance, mean that the disorder must have existed *at* birth, and continuously since birth, until at least the present moment? If so, would such a definition even apply to autism? To answer, one might point to the fact that there is no shortage of research highlighting the relevance of perinatal factors to the onset of autism,^18^ with even the birth process itself being highlighted as relevant.^19,20^ If one disregards the potential objection that such research can only tell you what goes on in relation to a group of people, and nothing about whether any of these factors were relevant to the genesis of autism in a specific individual, one could make a reasonable case that autism was a disorder that existed at birth. There is also little disagreement that such impairments tend to go on for a long time. Yet, the situation is arguably not quite this clear. For a start, whether autism is always (or even generally) present at birth remains a matter of debate, with at least one view being that symptoms of autism emerge over the first 18 months of life, having *not* been present at birth.^21^ Furthermore, whereas functional impairment might in many cases be expected to be ‘lifelong’, certainly some people with autism can show functional improvement either over time or in response to certain interventions or environments.^22,23^ Indeed, a recent review by Whiteley et al^24^ has questioned whether autism is a condition that will be lifelong for everybody with the diagnosis.

These issues suggest that ‘lifelong’, if it is to apply to autism, can therefore only be meaningfully read in broader terms – perhaps implying a condition that is rooted in early developmental issues, showing a consistent impact throughout the person's life, which perhaps varies in the quality and degree depending on the precise life circumstances and developmental stage. Alternatively, one might consider ‘lifelong’ to mean simply that the condition has continued to exist from the period of onset – whether that be birth, early childhood, adolescence or adulthood – until the present moment. Nobody would disagree that a broader definition like this could be applied to autism. However, of course, ‘lifelong’ could then apply equally well to plenty of other conditions. In making this point, one might reasonably note that many of the same perinatal factors implicitly reasoned as evidence of autism's ‘lifelongness’ might equally be applied to other conditions. For instance, personality disorder is clearly influenced by the experience of perinatal risk factors,^25^ leading to neurobiological vulnerabilities that, combined with the effects of early childhood adversity, lead to functional impacts across the lifespan;^26^ similar arguments could be made for schizophrenia.^9,27^ Moreover, although the purpose of this article is not to present a case that either disorder is indeed a ‘lifelong’ condition or not, both conditions have been described as ‘lifelong’ in multiple scholarly works (crudely, for instance, a ‘Google Scholar’ search conducted on 28 April 2023 returned 367 results for ‘schizophrenia is a lifelong’ OR ‘schizophrenia is a life long’), and if ‘lifelong’ were to be interpreted as simply a stronger synonym of ‘long term’, one would not need to look far to identify clinicians and academics who agreed with such a conceptualisation.

Could one reach a definition of ‘lifelong’ that included only autism but excluded these other conditions? Unfortunately, the rather philosophical questions about the points at which a disorder or condition begins and ends are probably unanswerable, at least in a way that provides legal clarity. Regardless, one either seems forced to read ‘lifelong’ in a way that could arguably exclude autism, or in a way which could arguably include plenty of other conditions. In these circumstances, it is reasonable to argue that the addition of this adjective does little to help clarify to whom the definition of legal autism applies.

## Would responsible clinicians be expected to apply the definitions pragmatically, focusing on the accepted clinical meaning?

An arguably more obvious reason that one might give for dismissing these concerns would be to rely on the fact that clinicians will ultimately make sensible and pragmatic decisions in practice, however the terms are legally defined. In this respect, it is certainly hard to imagine any responsible clinician actually making a case that a patient met the legal definition of autism if their clinical opinion was that their condition was better characterised by a clinical diagnosis of personality disorder or schizophrenia. Similarly, it is hard to imagine that a responsible clinician who believed a patient's difficulties were best characterised by a clinical diagnosis of autism would not conclude that the legal definition was also met. From this perspective, it would be understandable to argue that the concerns expressed thus far are somewhat overblown.

However, such an argument misses the exclusionary power of the definition, which becomes particularly relevant in the context of any challenge to a detention under s.3 (e.g. by way of a mental health tribunal (MHT) or hospital manager's hearing). It is important also to reiterate the earlier argument that ‘clinical autism’ and ‘legal autism’ are not the same thing, with the latter being a much broader concept that probably subsumes the former within it. In this light, one must consider that it is not the responsible clinician who will be making these arguments but legal advocates and clinicians instructed by those advocates representing the patient. In the case of a MHT, for instance, it is not the responsible clinician's views on clinical nosology that will carry weight, or their opinions on ‘clinical autism’, but the extent to which they can demonstrate that the legal criteria for detention are met. Given that the burden of proof is effectively on the detaining authority^28^ to demonstrate this on the balance of probabilities, it does not seem impossible that a proactive legal advocate could advance an argument that aspects of a person's presentation were characterised by legal autism even if this went far beyond whatever clinical yardstick of autism the responsible clinician might adopt. Such an argument could be quite powerful if combined with an assessment conducted by an independent clinician who advanced an argument in terms of the legal definition of autism only (Is it lifelong? Is it a developmental disorder of the mind? Does it affect how the person perceives, communicates and interacts with others?) and potentially even more compelling if the argument included reference to features that did at least overlap with typical clinical characteristics of autism. This becomes easier to imagine when one considers that core features of autism do indeed intersect with features of other conditions; for instance, impairments in emotional processing may be not dissimilar to those identified in psychopathic and narcissistic personality presentations – a point considered in more detail in our previous article.^1^ In such circumstances, a responsible clinician might be tempted to simply focus their argument on the fact that ‘psychiatric disorder’ was present and suggest the question about autism was irrelevant. However, this approach would probably be challenged, because the ‘psychiatric disorder’ concept is defined primarily by what it is not (i.e. legal autism or learning disability) rather than any other definitional features. Thus, given a question of legal autism being raised, any clinician seeking to demonstrate that psychiatric disorder *was p*resent would need to first establish that legal autism *was not*.

## Would MHTs be expected to apply the new definitions pragmatically?

Perhaps, of course, one might argue that MHTs may also respond pragmatically to the situation and in practice apply a definition of ‘legal autism’ that is more closely connected with the clinical definition. In particular, this might be through the MHT showing pragmatic deference to the responsible clinician's views on mental disorder and the relevant diagnosis. However, one must also remember that since the 2007 amendments, MHTs will have had very little business considering arguments about ‘mental disorder’ itself, given the breadth and scope of the definition (s.1(2)), with successful challenges being much more likely to be delivered against the requirements of ‘nature’ or ‘degree’ (s.2(2)(a)). When significant consequences are attached to the presence or absence of such a broad definition, there is good reason to believe that the nature of such arguments may change. Indeed, one might reflect that prior to the 2007 amendments, the relatively minor differences imparted by the categorisation of mental disorder (e.g. s.45A disposals could only be considered for people categorised as having ‘psychopathic disorder’) did indeed lead to legal challenges on the basis of the categorisation applied, e.g. *R v Staines*.^29^ The fact that people with autism often present with other problems that overlap with features of autism,^30,31^ where obvious arguments about which disorder might be creating a need for detention, only further muddies the picture.

## Do the existing exceptions to ‘mental disorder’ create a suitable precedent for an exception based on autism?

There is one further potential point that might be anticipated: the argument that exceptions have been carved out without issue in the current Act, in particular for learning disability (s.1(4)) and ‘dependence on alcohol or drugs’ (s.1(3)). One might argue, therefore, that adding autism as an exception is simply extending the existing framework of the Act. It is indeed the case that for most purposes (in the case of learning disability, s.1(2B)) and all purposes (in the case of alcohol or drug dependence), these conditions are carved out of the definition of ‘mental disorder’. However, in both cases, the definitions of the excluded disorders are substantially narrower than the proposed concept of legal autism and are also more directly and specifically linked to all of the key elements of the associated clinical construct. One could not effectively argue that ‘dependence on alcohol or drugs’ was the same thing as a personality disorder, nor that any of the other conditions identified were ‘a state of arrested or incomplete development of the mind which includes significant impairment of intelligence and social functioning’ (the existing definition of learning disability).However, even given these narrow parameters, the Code of Practice needs to give guidance on the operation of these exclusions in practice, for instance, highlighting that mental health conditions which arise as a consequence of dependence do remain within the scope of the definition of mental disorder. One can imagine that the guidance that would be necessary in respect of the proposed definitions would need to be exponentially longer.

## Potential solutions

How might the draft bill solve these potential issues without watering down the ambition to reduce restrictive interventions and detentions for people with autism? One might imagine a potential solution in amending the definition of legal autism such that it more closely resembled clinical autism or was perhaps narrower in scope. However, this is practically challenging in the other direction – turning clinical problems into legal definitions is far from easy; even legal definitions of ‘death’ have not been without discussion,^32^ and it is very hard to see how this could be achieved without causing further difficulties. Indeed, a key amendment in the 2007 changes was to (almost) abolish the use of diagnostic categories, a practice echoed also within the Mental Capacity Act (2005).

Another approach might be to use statutory guidance – possibly the Code of Practice – to indicate how autism is to be assessed and defined (and by whom) and, perhaps, how it is to be separated from ‘psychiatric disorder’ in practice. However, answering these questions is also likely to be complex, and in any case this would still arguably not solve the core issues caused by having such a broad statutory definition of autism.

A much bolder approach (in the other direction) would be to attempt to move much more strongly towards identification of separate conditions or presentations that would create a basis for detention. These could still be combined with a broader requirement for ‘mental disorder’ (perhaps broadened further to ‘mental disorder or developmental disorder of the mind’) if necessary but could, for instance, include in somewhat plainer English the actual scenarios which an admission was seeking to prevent, e.g. ‘suicide or self-harm risk’ or ‘harm in the context of a psychotic episode’. However, this would be an enormous change to the status quo and would require significant ethical, social and clinical input to avoid further unintended consequences and ensure all ‘domains’ could be reliably identified and differentiated.

An arguably more immediately workable solution might be to apply an additional ‘limiter’ to the definition of legal autism, perhaps something similar to that currently adopted for learning disability (i.e. ‘abnormally aggressive or seriously irresponsible conduct’). Although there remains subjectivity within these terms, and they are not welcomed in all quarters,^33^ they are at least focused on behavioural aspects that should directly relate to risk and would in practice mean that the concerns about the broad scope of legal autism would be moot for people with a condition other than autism who demonstrated such behaviour (of course, for those with the other condition who didn't meet this qualification, all the concerns raised above would continue to apply).

A final option might be to dispense with the effort to introduce a legal definition of autism and instead retain the broad definitions outlined in the current legislation. This would have the added advantage of making the introduction of a potentially unhelpful new category of ‘psychiatric disorder’ unnecessary (terminology which arguably implies medical causation of mental health conditions). In this scenario, one would then need to rely on the other changes in the bill, which aim to reduce unnecessary detentions for all people regardless of diagnosis, to achieve these aims for people with autism as well. Arguably, this option better fits the current evidence and understanding of mental health and of psychological, psychiatric and (neuro)developmental conditions, where heterogeneity within diagnostic categories and overlaps between them are arguably more the rule than the exception. Regardless, we hope that the present article stimulates further debate on this important issue and highlights the importance of a cautious approach in striking the balance between achieving protections and avoiding unintended consequences.

## About the author

**Peter Beazley** is Clinical Associate Professor at Norwich Medical School, University of East Anglia, Norwich, UK.
